# The Correlation Between Graft Size and Graft Failure in Hamstring Autograft Anterior Cruciate Ligament Reconstruction

**DOI:** 10.7759/cureus.55069

**Published:** 2024-02-27

**Authors:** Raed Y Abudaqqa, Amgad M Elsheoibi, Ali J Al Mas, Kariyal P Arun, Faris A Shaaban, Khalid A Aliessa

**Affiliations:** 1 Orthopaedics and Traumatology Department, Al Khor Hospital, Hamad Medical Corporation, Doha, QAT; 2 College of Medicine, Qatar University, Doha, QAT

**Keywords:** arthroscopy, graft failure, hamstring autograft, anterior cruciate ligament reconstruction, acl

## Abstract

Background: Previous studies have investigated various factors that contribute to graft failure in hamstring anterior cruciate ligament (ACL) reconstruction. However, there is debate about the potential advantages of increasing graft diameters beyond 8 mm.

Methods: In this retrospective cohort study (level of evidence III), we investigated whether increasing graft sizes beyond 8 mm diameter showed any advantages in reducing the risk of graft failure. We utilized univariate Kaplan-Meier analysis and Cox proportional hazard (PH) regression models to compare the risk of failure in the different patient groups. Mutual adjustment was performed for age, gender, body mass index (BMI), and graft strands. Graft sizes ranging between 8 and 10 mm were assessed for their association with graft failure, alongside examining the impact of graft strands, age, and BMI on graft failure.

Results: A total of 487 patients underwent hamstring autograft ACL reconstruction at our hospital between January 2016 and December 2020. Our analysis indicated that among patients undergoing hamstring autograft ACL reconstruction, the risk of graft failure was 1.64 times higher for patients with a graft size between 8.5 and 9 mm (95% CI 0.36-7.43, p=0.483) and 2.19 times higher for patients with a graft size between 9.5 and 10.5 mm (95% CI 0.42-11.31, p=0.384) compared to those with an 8 mm graft. However, there was weak evidence against the null hypothesis.

Conclusion: These findings suggest that there is no benefit to increasing graft sizes past 8 mm and that other factors, like surgical technique, should be considered when assessing the risk of graft failure in patients undergoing ACL reconstruction.

## Introduction

Anterior cruciate ligament (ACL) tears are a common injury worldwide, affecting up to 75 per 100,000 person-years, especially among young individuals who engage in contact sports [1]. Surgical reconstruction of the ACL is often necessary to restore functional stability and prevent degenerative changes in the knee joint [2,3]. Surgeons use a variety of graft types, each with its advantages and disadvantages, to perform the reconstruction. Despite advances in surgical techniques and extensive research, there is still ongoing debate regarding the ideal graft diameter for each patient. Hamstring autografts are commonly used for ACL reconstruction (ACLR), often in a four-strand or five-strand configuration. Studies have reported that the mean graft diameter of hamstring autografts ranges from 7.7 mm to 8.5 mm [4,5].

Biomechanical studies have shown that hamstring autografts have an initial strength that exceeds that of bone-patellar bone (BPB) autografts, making them a viable option for ACLR [6,7]. They are also considered superior as their harvesting does not compromise the integrity of the extensor mechanism of the knee and is associated with a lower risk of postoperative knee pain [7-9]. However, the strengths of these grafts depend on their diameter, and there is an argument among experts about what size is necessary for optimal mechanical functioning. Moreover, surgical experience and magnetic resonance imaging (MRI) studies show significant discrepancies in hamstring size among the population [10,11]. Some biomechanical studies suggest that larger graft sizes are more beneficial as they account for potential strength reduction during graft healing [12]. Previous studies have also shown that smaller graft sizes are correlated with an increased risk of failure in these patients [12,13]. Other studies have also shown that younger age may be associated with graft failure. For example, Shelbourne et al. found that younger patients were at an increased risk for graft failure and were less tolerant of recurrent instability, which may result in higher revision rates in younger patient populations [14].

In this study, we aimed to investigate whether increasing graft sizes past 8 mm reduces the risk of graft failure in hamstring autograft ACLR patients. We also aimed to investigate the correlation between demographic variables (age and body mass index (BMI)) on the risk of graft failure.

## Materials and methods

Study design and patient population

This is a retrospective cohort study (level of evidence III) of previously collected medical records of ACLR patients. After our institution's approval, we retrospectively reviewed the medical records of all patients who underwent autograft hamstring ACLR at our hospital (Al Khor Hospital, Qatar) between January 2016 and December 2020. ACLR was performed by three senior consultant surgeons, and all patients underwent the same surgical technique, rehabilitation protocol, and follow-up.

Surgical technique and rehabilitation

All patients underwent a standardized arthroscopic single-bundle ACLR procedure, performed by a senior consultant. To harvest the hamstring graft, we made an approximately 5 cm longitudinal skin incision, starting three fingers distal to the knee articular line and 2 cm medial to the tibial tubercle. Gracilis and semitendinosus tendons were harvested with a closed tendon stripper. The graft was then prepared by an assistant surgeon as three, four, five, or six strands depending on the final graft size and shape, with a measurement of 8-10.5 mm graft diameter and a minimum of 80 mm for graft length. Femoral graft fixation was done using an endobutton (Smith & Nephew endoscopy), and tibial fixation of the graft was done using 25 mm absorbable interference screws or with/without metal staples, with the knee at 30 degrees of flexion.

We performed knee arthroscopy via anteromedial and anterolateral portals. The femoral condyle and tibial aspects were cleaned and well-prepared. We detected the femoral tunnel position at the center of the femoral footprint, with the knee flexed 120 degrees. Using a 2.4 mm guide wire, we identified the femoral tunnel and then drilled it with a 4.5 mm cannulated drill pit. The femoral tunnel was measured and correctly drilled according to the size of the graft, and approximately 5 mm of the margin of the lateral femoral cortex was preserved intact. The tibial tunnel position was identified on the tibial footprint of the ACL, in line with the anterior horn of the lateral meniscus and tibial spine. By 2.4 tibial guide wire, the tibial tunnel was drilled. We then passed a threaded suture across the femoral tunnel to the tibial tunnel through the knee joint. The prepared hamstring graft was delivered through the tibial and femoral tunnels using the already passed threaded suture. Cycling 20 times and then knee flexion to 30 degrees, the tibial side graft was fixed with a bioabsorbable screw over a guide wire [15,16].

The operations for all patients were done at the daycare units, and they were discharged home the same day. Patients started physical therapy (three sessions per week) immediately after surgery. Rehabilitation focused on full range of motion (ROM) of the knee and quadriceps-hamstring muscle strengthening during the first six weeks, with weight bearing using axillary crutches and the use of knee braces until suture removal. Returning to sports was allowed six months post-surgery.

Inclusion and exclusion criteria

To be included in the study, patients had to have undergone ACLR with an autograft hamstring and femoral graft fixation using endobutton (Smith & Nephew). The minimum follow-up time after the surgery was set at one year, and complete patient demographics and surgical and follow-up data had to be available on medical records. The study also included only primary ACLR cases. On the other hand, patients who underwent revision ACLR, had incomplete medical records, or experienced graft failure due to high-energy trauma were excluded from the study. These criteria were put in place to ensure the accuracy and validity of the study results.

Data collection and variables

Data collected from hospital electronic records included patient demographics (gender, BMI, age), surgical data (operative time, associated knee injury, graft size, number of strands, type of graft fixation), and clinical outcome (signs of instability via Lachman, anterior drawer tests, pivot shift test, MRI result, and graft failure). The primary study outcome was graft failure which is defined as recurrent knee instability, ACL revision, and MRI diagnosis.

Statistical analysis

The data was analyzed using Stata Statistical Software: Release 17 (2021; StataCorp LLC, College Station, Texas, United States). Graft sizes were initially reported in 0.5 mm increments but were later categorized into 8, 8.5-9, and 9.5-10.5 to provide more meaningful results. BMI was calculated using weight and height data and then categorized into normal (≤25), overweight (25-30), and obese (≥30). Age was categorized into quartiles: <25, 26-30, 31-35, and 36-55. Univariate Kaplan-Meier survival analysis was used, along with the Cox proportional hazard (PH) regression model, to compare the risk of failure among the different graft sizes. The link test was used to assess the proportionality assumption of the Cox PH regression model in Stata. Models were adjusted for age, gender, BMI, and graft strands. A hazard ratio (HR) with a 95% confidence interval (CI) was used as an estimate of the risk of failure.

Ethical approval

This study was carried out according to the ethical principles of the Declaration of Helsinki (World Medical Association 2013) [17]. Participant data utilized in this study was de-identified, and all members of the research team were trained in ethics and good clinical practice before the commencement of the study. The study was approved by the Medical Research Center at Hamad Medical Corporation (approval number: MRC-01-23-289).

## Results

Patient characteristics

A total of 487 patients underwent hamstring graft ACLR at our hospital (Al Khor Hospital, Qatar) between 2016 and 2020. The median follow-up period for these patients was 17 months. Only 18 patients experienced graft failure following ACLR. The mean age of the participants was 30.6(±7.2), and the vast majority of them were male (99.6%). Most patients were overweight (41.8%). Regarding surgical details, the majority of patients had graft strands of five or fewer (85.9%) and graft sizes of 8.5-9 mm (55.6%). Around 58.2% of the operated ACLRs were on the right knee (Table 1).

**Table 1:** Baseline characteristics of patients who underwent hamstring graft ACLR at Al Khor Hospital between 2016 and 2020 The data is presented in the form of mean±SD or n(%) IQR: interquartile range; SD: standard deviation; BMI: body mass index; ACLR: anterior cruciate ligament reconstruction

Factor	Level	Value
N		487
Follow-up (months), median (IQR)		17 (14, 29)
Failure	No	469 (96.3%)
	Yes	18 (3.7%)
Age (years), mean (SD)		30.6 (7.2%)
Sex	Female	2 (0.4%)
	Male	485 (99.6%)
BMI (kg/m^2^)	Normal	144 (29.6%)
	Overweight	222 (45.6%)
	Obese	121 (24.8%)
Laterality	Left	204 (41.9%)
	Right	283 (58.1%)
Graft strands (strands)	3	7 (1%)
	4	29 (6%)
	5	380 (78%)
	6	71 (15%)
Graft size (mm)	8	104 (21.4%)
	8.5-9	279 (57.3%)
	9.5-10.5	104 (21.4%)
Staple	No	326 (66.9%)
	Yes	161 (33.1%)

Correlation between graft size and strands with risk of failure

**Figure 1:**
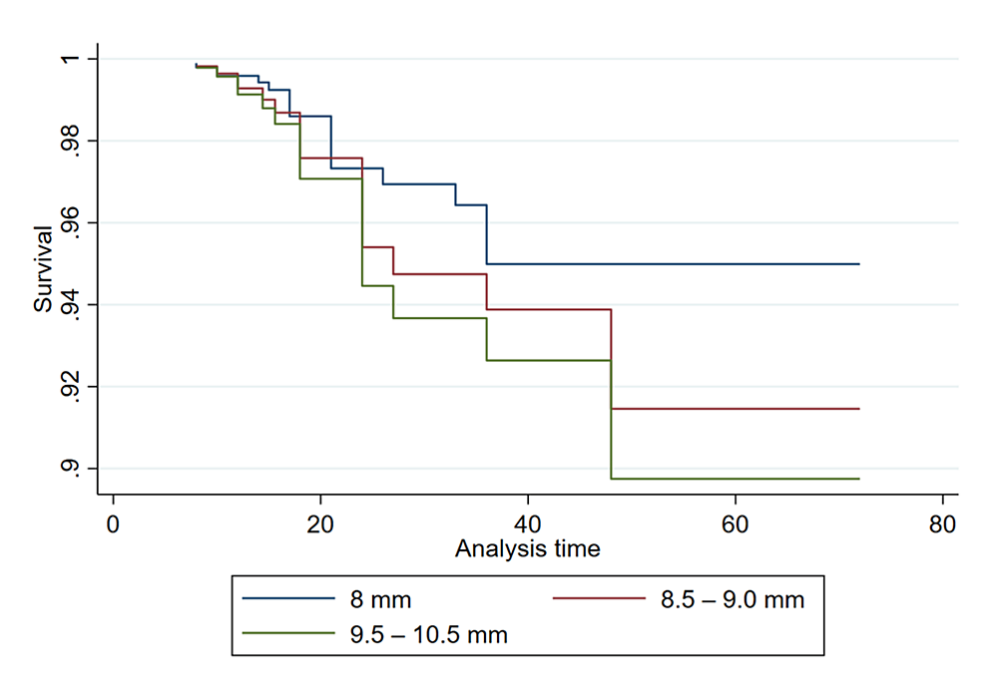
Survival function plotted for adjusted Cox PH model stratified by graft size PH: proportional hazard

**Figure 2:**
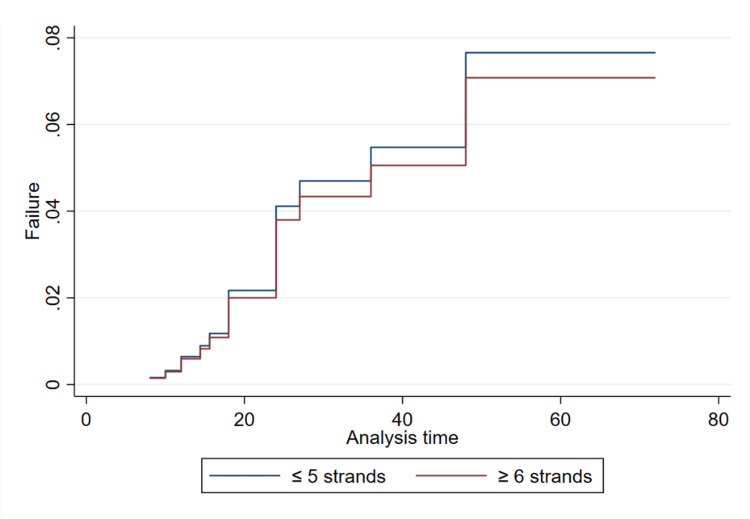
Failure function plotted for adjusted Cox PH model stratified by graft strands PH: proportional hazard

The results of the Cox PH regression are summarized in Table 2. The results revealed graft sizes greater than 8 mm may be associated with an increased risk of overall graft failure (Figure 1), although the effect did not reach statistical significance in our adjusted analysis. Specifically, the unadjusted analysis showed that the risk of graft failure was 1.64 (95% CI 0.36-7.43) times higher for patients with a graft size between 8.5 and 9 mm and 2.19 (95% CI 0.42-11.31) times higher for patients with a graft size of ≥9.5 mm, compared to the reference group (8 mm). After adjusting for age, gender, and BMI, the risk of graft failure was 1.74 (95% CI 0.37-8.13) for the 8.5-9 mm group and 2.10 (95% CI 0.39-11.23) for the ≥9.5 mm group, respectively. There was no association between the number of strands and failure in patients undergoing hamstring graft ACLR. Unadjusted analysis revealed that graft strands ≥6 had 1.22 (95% CI 0.40-3.72) times higher risk of failure compared to the ≤5-strand group; however, the results were not statistically significant and disappeared after adjustment (HR: 0.91, 95% CI 0.29-2.92). This can also be seen visually in the modeled Cox PH regression (Figure 2).

**Table 2:** Results of the Cox PH regression model BMI: body mass index; HR: hazard ratio; CI: confidence interval; PH: proportional hazard

Variable	Sublevel	Unadjusted		Overall adjusted	
		HR (95% CI)	P-value	HR (95% CI)	P-value
Graft size (mm)	8	-	-	-	-
	8.5-9	1.64 (0.36-7.43)	0.520	1.74 (0.37-8.13)	0.483
	9.5-10.5	2.19 (0.42-11.31)	0.350	2.10 (0.39-11.23)	0.384
Age	≤25	-	-	-	-
	26-30	0.54 (0.14-2.18)	0.391	0.53 (0.13-2.17)	0.377
	31-35	1.25 (0.42-3.75)	0.685	1.26 (0.41-3.86)	0.690
	36-55	0.40 (0.08-1.99)	0.262	0.40 (0.08-2.15)	0.288
BMI	Normal	-	-	-	-
	Overweight	1.02 (0.38-2.74)	0.973	1.06 (0.38-2.96)	0.916
	Obese	0.39 (0.08-1.87)	0.238	0.37 (0.07-1.81)	0.217

Correlation between age and BMI with risk of failure

The results of the Cox PH regression model suggest that there is no significant association between age and graft failure in hamstring graft ACLR. The adjusted HRs and corresponding 95% CIs for age groups of 26-30 (HR 0.54, 95% CI 0.14-2.18), 31-35 (HR 1.25, 95% CI 0.42-3.75), and 36-55 (HR 0.40, 95% CI 0.08-1.99) were not statistically significant, indicating that the risk of graft failure was similar across these age groups when compared to the reference group of ≤25. Similarly, for BMI, there was no association between overweight and graft failure in hamstring ACLR in both the unadjusted (HR 1.02, 95% CI 0.38-2.74) and adjusted (HR 1.06, 95% CI 0.38-2.96) models. For the obese category, the HR suggests that obesity may be associated with a lower risk of failure in ACLR; however, this was not statistically significant and is probably due to reduced activity.

## Discussion

In this study, we investigated the correlation between various factors and the risk of graft failure in hamstring ACLR. The increase in graft diameter greater than 8 mm and number of strands did not appear to have a significant correlation with graft failure. Age and BMI were also not significantly associated with the risk of graft failure.

Although our study did not identify a statistically significant difference in graft failures in patients with graft sizes of 8 mm or greater, this is not the first study to report such findings [18-22]. A study by Park et al. on 296 patients who underwent hamstring autograft ACLR showed that there were no statistically significant differences between failure rates in increments of 0.5 mm graft sizes between 5 and 9.5 mm. When they classified patients into <8 mm and ≥8 mm, they found that failure rates were higher in the first group of patients [22]. Another study by Kamien et al. on 98 patients who underwent hamstring autograft ACLR and were followed for at least two years showed that there was no association between graft diameters and failure rates [20]. Schlumberger and colleagues also investigated this same association in over 2448 cases and found no statistically significant differences between the different graft sizes and failure rates even after categorizing the patients into ≤8 and >8 mm [23].

Marchand and colleagues investigated the association between graft diameters and laximetric outcomes in hamstring ACLR. In their study, they had no patients with graft diameters smaller than 8 mm, and they reported that increases in graft diameters over 8 mm provided no improvement in laximetric outcomes in ACLR [21]. To date, the largest study to assess the association between graft size and failure in hamstring autograft ACLR was a prospective study by Inderhaug et al. They also reported that graft size and BMI were not independent risk factors for ACL graft failure or surgical revision [19]. The study included a large sample size and had a long-term follow-up, which strengthened the reliability of their findings. Therefore, their results provide valuable insights into the factors that may influence the success of ACLR surgery.

The findings of these studies support the idea that graft size may not be an independent predictor of failure in ACLR surgery and, hence, should not be the main concern for a surgeon. Ideally, the hamstring autograft utilized for ACLR should be at least 8 mm in diameter, and several methods have been published in the literature to achieve this. There is no need for harvesting and adding more tissue to the graft to increase its diameter past this as the data suggests that it provides little to no benefit. However, it is still important to consider the potential influence of graft size and age on ACL graft failure. This is because multiple studies have reported a correlation between smaller graft size and an increased risk of graft failure, especially in younger patients. For instance, Mariscalco et al. reported that small graft size is a predictor for poor outcomes and a higher rate of failure in hamstring ACLR [24]. Similarly, Magnussen et al. found that smaller graft thickness of hamstring autograft (8 mm or less) in younger patients (under 20 years of age) is associated with higher revision rates [13].

There are several potential explanations for the lack of significant associations in our study. One possibility is that our sample size was not large enough to detect a true difference. This is understandable as the occurrence of failure is relatively low. Finally, it is possible that other factors, such as surgical technique, postoperative rehabilitation, or patient activity, may have a greater impact on graft failure than graft size or age. Hence, despite the lack of significant association in our study, it is still important for surgeons to consider graft size and patient age when selecting a graft type for ACLR. While our study did not find a significant difference, previous research has suggested that younger patients may be at higher risk for graft failure and this information should be taken into account when making treatment decisions [13,25]. Additionally, further research is needed to better understand the relationship between graft size, age, and ACL graft failure and to cast the spotlight on additional factors that could influence ACL graft failure.

Our study has several limitations. The follow-up period is relatively short, as this study focuses on early graft failure. However, graft failure can occur well after the follow-up period included in this study. Another limitation is the retrospective nature of our study. We were also unable to control for other possible causes of failure, including activity levels and tunnel malposition. This was mainly due to the lack of data on this. Finally, we lack validated patient-oriented outcome scores and postoperative activity-level data that could yield more insight into the influence of graft size on the outcome. Given these limitations, caution should be exercised when considering the outcomes of this study. Further work is needed to confirm and clarify these results.

## Conclusions

This study did not find a statistically significant association between graft size, number of strands, age, BMI, and the risk of graft failure in hamstring ACLR. Further research is needed to better understand this correlation. Overall, other factors may have a greater impact on the success of ACLR, and careful consideration of patient characteristics and surgical techniques is crucial for optimal outcomes.
